# Mr. Jordan W. Lambert

**Published:** 1889-03

**Authors:** 


Mr. Jordan W. Lambert, President of the Lambert Pharmacal Company, died early in January of the present year, at his home in St. Louis. Mr. Lambert was a genial gentleman, personally well known to thousands of American physicians, who yearly met him at the meetings of the American Medical Association, and who will sincerely deplore his sudden and early death.

